# A Challenging Sexsomnia Seen as a Deceptive Case of Depression

**DOI:** 10.1192/j.eurpsy.2023.2344

**Published:** 2023-07-19

**Authors:** J. Brás, M. Meira e Cruz, C. Teixeira, R. Andrade, A. P. Costa

**Affiliations:** 1 Department of Psychiatry and Mental Health, Centro Hospitalar Tondela Viseu, Viseu; 2 Sleep Unit, Centro Cardiovascular da Universidade de Lisboa, Lisbon School of Medicine; 3 European Sleep Center, Lisbon, Portugal

## Abstract

**Introduction:**

Sleep related sexual behaviors or sexsomnias are unconscious behavioral activities that occur during sleep (e.g. parasomnias). Behaviors could range from sexual vocalizations, orgasms, sexualized movements, masturbation, or full sexual intercourse with a subsequent amnesia. Early epidemiological studies showed a prevalence of 7.1%, with a male predominance. While intended as a rare condition, leads to important physical and psychological consequences for both the patient and their bed partner. For our knowledge this is the first case of sexsomnia reported in Portugal.

**Objectives:**

To report the clinical and psychosocial impact of a Sexsomnia case in a young woman which was misdiagnosed with depression.

**Methods:**

Patient´s clinical files consultation and literature review using Pubmed^â^ and the keywords: *sexsomnia.*

**Results:**

A 18-year-old female referred to a psychiatric consultation to be assessed and treated from a diagnostic of depressive disorder. This was a young woman with a previous history of sleepwalking during childhood, with no recurrent episodes since adolescence. A familiar positive history for sleepwalking was confirmed (mother). She reported the beginning of her sleep related sexual behavior six months before the consultation, conflicting with the moment in which she started pharmacological therapy for Chron Disease, diagnosed at that time.

After she slept with her boyfriend, she was told by him about the recurrence of masturbatory activity during sleep. These episodes were told to occur as often as 1 to 2 times a night, shortly after falling asleep, with posterior amnesia for the event.

As for medical or psychiatric history, only Chron’s disease is highlighted, being under control with azathioprine. Likewise, he took 1mg of melatonin/night.

Pittsburgh Sleep Quality Index at presentation was 7/21 and the STOP-Bang questionnaire revealed a low risk of Obstructive Sleep Apnea.

A Type I Polysomnographic study was performed revealing decreased sleep efficiency and fragmented sleep presenting an alternating cyclic pattern. The existence of significant respiratory events during sleep, as well as periodic movements, was excluded.

Cognitive behavioral therapy by means of highlighting the need of improvement on sleep hygiene measures was prescribed and the dose of melatonin was increased up to 3mg. Despite the good clinical response, the patient discontinued the melatonin treatment mainly due to familiar and personal reasons and failed to comply with the prescribed hygienic measures, with a further worsening of the clinical condition.

**Conclusions:**

This particularly challenging case representing the emerging medicolegal issues and psychosocial aspects related with the still poorly understood sleep disorders like sexomnia, shows up how much awareness is required from psychiatric team members to better assist and refer patients, promoting both an assertive diagnostic and an effective management.

**Disclosure of Interest:**

None Declared

